# Explaining the Social Acceptance of Renewables through Location-Related Factors: An Application to the Portuguese Case

**DOI:** 10.3390/ijerph18020806

**Published:** 2021-01-19

**Authors:** Lígia M. Costa Pinto, Sara Sousa, Marieta Valente

**Affiliations:** 1 NIPE and EEG, University of Minho, 4710-057 Braga, Portugal; mvalente@eeg.uminho.pt; 2 ISCAC/Coimbra Business School and CERNAS, Polytechnic Institute of Coimbra, 3040-316 Coimbra, Portugal; ssousa@iscac.pt

**Keywords:** renewable energy sources, wind power, hydropower, solar photovoltaic, energy production, social acceptance, location-related factors

## Abstract

The public perception of renewable energy sources is generally positive, due to their role in air pollution and CO_2_ emission mitigation policies. However, there are local environmental detrimental effects, and empirical evidence is not consistent as to the support of local communities. In the present paper, we analyse the antecedents of public generic perceptions of renewables grounded on objective location-related factors. Personal location-related factors can originate in the involvement of individuals with renewable energy sources. Regional location-related factors concern the importance of the renewable energy source in the district of residence and in relation to other renewables. We implement a questionnaire on public perceptions of renewable energy sources by the general population in mainland Portugal and complement respondent-level responses with renewable energy district information. Regression analysis shows that these objective location-related factors, both personal and regional, help explain public perceptions of renewables and thus we find empirical support for the proposed approach. These results can inform and guide policymakers in tackling future social acceptance issues of renewable energy policies towards lower carbon emissions and less polluting energy production.

## 1. Introduction

Renewable energy sources (RES) have been a fundamental part of climate change policies. The use of RES in electricity production has been proposed as a substitute for thermal electricity production powered by fossil fuels to lower carbon emissions and reduce air pollution and associated health problems [1]. In addition, the use of RES increases energy security and insures against crude oil price volatility. Moreover, according to the 2016 World Energy Outlook [2], energy demand is expected to increase by 30% until 2040, and most of the increase is expected to be satisfied by RES. Consequently, many communities will be affected by the decisions regarding the siting of energy producing facilities. In the case of RES, siting decisions are conditioned on the location of the source of the energy. As the source of renewable energy is, in general, not mobile, as is the case with fossil fuels or natural gas, possible siting locations for RES facilities are limited (except for biomass power plants).

The substitution of fossil fuels by RES, such as wind, hydropower, solar and forest biomass, produces significant environmental and health benefits by reducing air pollution. However, the use of RES in electricity generation is not environmentally neutral: impacts on fauna, flora, soil fertility, landscape, cultural heritage, noise, and people displacement are some of the environmental and social adverse impacts associated with the operation of different renewables’ power plants (e.g., [3,4,5,6,7]).

Also noteworthy is the fact that renewables’ adverse effects are mostly local in scope, affecting mainly the well-being and quality of life of those residing in the local communities next to renewables’ power plants, while the benefits accruing from the substitution of fossil fuels by RES are global, benefiting the entire population. This is clearly a situation of environmental and energy injustice (e.g., Jenkins et al. [8]; Jenkins et al. [9]; Walker [10]). As stressed by Sovacool ([11], p. 15), “how we distribute the benefits and burdens of energy systems is preeminently a concern of any society that aspires to be fair”. This raises a key question about how the costs and benefits of energy production and consumption should be distributed. The fact is that social issues are often neglected in energy planning [12], resulting in some controversial situations, with significant discontent and opposition from local populations regarding the imposed proximity of some RES power plants.

The equity considerations raised have been addressed in the context of social acceptance theory. Social acceptance of renewable energy innovations is of utmost importance for the successful implementation of RES power plants and includes both socio-political and community acceptance [13]. On the one hand, the general public perception of the use of renewables is mostly positive. On the other hand, the empirical evidence on the perceptions of local communities is not consistent. In some cases, the evidence reveals negative opinions towards the installation of particular power plants in the vicinity of respondents’ residences [14]. RES power plants tend to be small, and dispersed over the territory, increasing the number of people exposed to their potentially detrimental effects (The fact that there are many RES power plants means they can potentially cause, although of different magnitudes depending on size, location and type of energy, nuisances to many local residents. It is beyond the scope of this study to explore how the nuisances caused (in terms of total impact or number of people impacted) would compare to a non-RES power plant—we thank an anonymous reviewer for pointing this out). This effect varies across sources. Specifically, dams for hydropower electricity production tend to be located in specific and remote places, exposing fewer people to impacts than other sources. However, when the projects are large-scale, they may cause many nuisances to the local population. Additionally, if wind power plants or solar photovoltaic plants are expected to match the same installed capacity as large dams, they tend to occupy a significantly wider area. On the contrary, smaller scale projects cause only minor nuisances.

We argue that these two dimensions of social acceptance of RES, namely socio-political and community acceptance, may potentially be affected by how individuals relate to RES because of their location in relation to RES power plants. We argue that individuals in a region with more installed renewable capacity will be more acquainted and involved with specific sources. This phenomenon will be reflected in more daily contact with the installation, as is the case of a more visible installation seen daily, and thus individuals’ opinion will reflect this increased contact. Additionally, regions where one energy source is more predominant than others will condition the individual’s relative perception of an energy source relative to those less predominant or absent. Additionally, it is likely that the higher involvement that individuals have with renewables in terms of their work experience or their closer contacts will also affect the perception of RES.

Previous studies have considered location-related factors as explaining perceptions of RES friendliness; however, they have focused on a specific region rather than on broader and more generalizable characteristics. Soliño et al. [15] put forth that economic and geographical characteristics of respondents’ regions can impact economic valuation, in the case of forest biomass. We propose in this study to consider a set of location-related factors that are directly linked to renewables, rather than to geographical characteristics per se. With these factors, we propose to explain differences in public perceptions of renewable friendliness, either through direct personal factors or through characteristics of the region. We test this approach with data from a case study in Portugal (mainland). With this study, we aim to enrich the empirical literature on determinants of public opinion towards renewables. More recently, several authors have acknowledged that observed public opinion often diverges when abstract perceptions are elicited or when the focus is on a specific real project (e.g., [16,17]). One strategy to bridge this gap has been to study generic perceptions but towards projects that are localized, albeit hypothetical. Our proposed approach is to focus on generic perceptions but attempt to explain them through location-related variables, thus providing an alternative strategy. We use respondent location information to understand if respondents are somehow involved with RES through employment or closeness to a power plant. Additionally, based on the location of the respondent, we can characterize the district of residence in the importance of renewables. These antecedents can thus be objectively and easily measured by decision-makers interested in understanding public perceptions and in addressing them through information campaigns.

Through a questionnaire of a representative sample of the Portuguese population, we elicited the perceptions of the general population towards selected RES, as well as factors related to the respondents’ work and residence locations, and direct/indirect personal involvement with RES. We focus on the three better known sources in Portugal, namely hydropower, wind power and solar-photovoltaic power. We then matched information on renewables in the district of residence for each respondent. Through regression analysis, we test a model to explain perceptions of friendliness of RES through location-related factors.

We indeed find that location-related factors have significant impacts on the perception of friendliness of renewables. The nature of this relation depends on the RES illustrating how specific RES setups from the perspective of the individual can condition the social acceptability of renewables in general.

In the next section, we present an overview of the main concepts in the literature that underlie the relation between individual perceptions towards renewables and location-specific factors, to justify our research question. In Section 3.1, we present the method used to measure the concepts and in Section 3.2, we develop the empirical study to test the research question, by briefly referring to the context of the case study used to test the model, namely the Portuguese case, and presenting an overview of the data used. The results are then presented in Section 4 and Section 5 concludes.

## 2. Literature Overview

Using RES for electricity generation has undeniable advantages over traditional fossil fuel sources, in particular in terms of air pollution and CO_2_ emissions. However, RES are not environmentally benign [18], especially for local communities. The installation and operation of electricity generation installations can have non-negligible impacts (e.g., [19,20]) and these vary across energy sources. In terms of broad categories of impacts, studies have documented the negative impact in terms of landscape intrusion, visual pollution, wildlife disruptions and biodiversity effects of windpower (e.g., [21,22,23]), hydropower (e.g., [24,25,26]) and solar photovoltaic energy (e.g., [17,27,28]).

Empirical studies on RES often find overall support for the use of renewables as opposed to traditional fossil fuels. However, when studies focus on specific RES projects, local opposition is observed, namely from the local communities living nearby [16,29]. This is indeed a key issue in the development of renewable energy policy. As stressed by Wüstenhagen et al. ([13], p. 2683), “there is one factor that can potentially be a powerful barrier to the achievement of renewable energy targets: social acceptance”.

A direct interpretation of local opposition to RES facilities relates to the so-called not-in-my-backyard (NIMBY) phenomenon [30]. NIMBY refers to the “protectionist attitudes of and oppositional tactics adopted by community groups facing an unwelcome development in their neighbourhood” ([31], p. 288). Often, empirical studies have focused on the relation between physical proximity and opposition to RES [32]. The fact is that there is empirical evidence that some renewable installations negatively affect reported life satisfaction of local residents [20] or produce non-negligible monetary valuations of nuisances experienced by locals [6]. In those cases, we can expect a negative impact on the local support concerning a local RES project and on the generic support for each RES. Aitken [33] underlines that public attitudes and responses to renewables should not be examined in order to mitigate potential future opposition, but rather in order to understand the social context of renewable energy.

NYMBYism is however not a straightforward concept in the literature, as some authors argue that local opposition depends on the symbolic interpretation of renewable energy facilities; as in one example in McLachlan [34] where a biomass plant can be interpreted as a “factory with chimneys” or a modern plant that produces green electricity; or in a case study of wind turbines reported in Firestone et al. [35].

According to Wüstenhagen et al. [13] the concept of social acceptance is multidimensional including socio-political acceptance, community acceptance and market acceptance. Therefore, the analysis of social acceptance, as an alternative to NIMBYism, comprises a broader perspective, interpreting opposition by local communities to the installation of new technologies as ‘place protective actions’ [36,37]. As such, these attitudes may be related to explanations of place attachment and place identity.

Local support for local RES projects is clearly dependent on the experience with the actual projects. On the other hand, global support, which is a more abstract perception, may also be shaped by the context of the individual in terms of RES, namely familiarity with renewables and how RES shapes place identity. We argue that it is not only local support but global support for RES that depends on the specific RES context of the respondent. 

The literature does not suggest a straightforward relation between location-related factors and local or global support for renewables. On the one hand, place attachment encompasses different spatial ranges from the home to the neighbourhood or to the city and region [38,39,40,41]. For example, Devine-Wright and Batel [42] find in the case of high voltage power lines that place attachment perceived at the national level, rather than local, increased the support for those projects. However, in terms of RES projects, most studies have found that when individuals exhibit high levels of place attachment at the local level, they tend to oppose those projects (e.g., [43,44]). Some authors (e.g., [34,35,45,46]) focus their analyses on place meaning and argue that it is the relationships that individuals establish with the place, or its elements such as landscape, that significantly determine individuals’ response to the installation of new renewable projects.

By contrast, community participation and empowerment at the planning stage help foster place attachment [47] and reduce local opposition to projects. A study in two rural Scottish communities found that new wind power projects engendered positive impacts on place identity due to the local development possibilities [48]. For other individuals in the same communities, the projects were seen as disruptions to place identity. It is therefore possible for the local context of RES of individuals to impact either negatively or positively their concept of place attachment, which in turn impacts their support for renewables.

In this research, we propose to study social acceptance for the case of different RES considering as antecedents objective location-related factors, either personal factors or regional factors. Particular attention is devoted to the regional intensity in terms of renewables, which will underlie the contact individuals have with RES. On the one hand, we argue that the more personal contact and self-involvement individuals have with RES (personal factors), the more acceptable the RES is. Furthermore, a more general acquaintance with RES due to intensity in the district can also reinforce this acceptance. On the other hand, it is possible that negative experiences with local RES translate into negative perceptions, thus dampening support for RES.

To test this approach, we use data from a case study in Portugal [19], where the perception of environmental friendliness of different RES (hydropower, wind power and solar photovoltaic power) was elicited through a questionnaire. We then collect data on location-specific factors, either through regional statistics on RES for regional factors, or from the questionnaire responses in terms of personal factors.

Previous studies in Portugal find positive public attitudes towards RES technologies [49] with survey respondents mostly acknowledging local economic benefits from projects rather than global environmental benefits. In particular local residents who benefit from the RES directly, for example in terms of employment express support for the RES installation [50]. Until recently, studies focusing on Portugal had neglected local environmental and social adverse impacts, but energy-specific empirical studies have drawn attention to their acknowledgment both by the local population and national residents (e.g., [51,52]). 

## 3. Materials and Methods

### 3.1. Methodology

The aim of this research was to explore whether the public acceptance of RES is influenced by objective location-related factors, either directly through personal factors or indirectly through regional characteristics related to renewables. We designed a questionnaire to be administered through personal interviews (the questions analysed in this paper are included as Supplementary Material). Literature analysis and expert consultation were conducted to identify key features of RES perceptions in the general population, as well as focus groups and think-aloud sessions, so as to ground the questionnaire in insights from qualitative data.

To capture the perception about RES, there was a question about the degree of friendliness of each renewable using a 5-point Likert scale (value 1 and 2 corresponded to the perception of negative effects, namely 1: *very unfriendly* and 2: *somewhat unfriendly*; value 3 to indifferent and values 4 and 5 to positive effects, namely 4: *somewhat friendly* and 5: *friendly*) (variable *perception of friendliness of RES*). So as not to force a response, this question included the possibility for respondents to state that they did not have an opinion, which allowed us to identify and exclude those individuals from the analysis, which would not have been possible if a forced opinion had been elicited.

The questionnaire also included socio-demographic questions as background questions. Additionally, we elicited the perception of respondents on environmental and RES related questions. Concerning environmental problems, respondents were asked to indicate the three main problems facing the country at the time of the questionnaire.

To account for location-related factors through personal factors, we included in the questionnaire two questions. First, respondents are asked to indicate whether they, a family member or an acquaintance worked at the time of the questionnaire or worked in the past in the renewable energy sector. This question captures a potential personal gain from the presence of the renewable sector in the district of residence. We constructed the variable involvement as a categorical variable to indicate if the respondent would, through that channel, directly or indirectly benefit from RES.

Second, a stronger presence in the district of residence may imply that individuals come into contact with the renewable energy sector infrastructure on a daily basis. To capture this effect, there was a question as to whether respondents saw a RES power plant from home, work or during the daily commute (categorical variable visibility).

Location-related factors can also originate from regional characteristics of the respondent’s district of residence. We aimed to account for the presence and importance of renewables, so we constructed two variables from statistical databases. One factor related to the importance of the specific renewable under appreciation in the region. A categorical variable was created which captured whether each RES individually was the renewable source with the highest installed capacity in the region (variable *preponderance of wind/hydro/solar photovoltaic power*); for example, the variable *preponderance of solar photovoltaic power* takes value 1 if solar photovoltaic was the source with highest installed capacity in the district, and was thus the most salient renewable in that particular district.

Additionally, we wanted to capture the importance of the region in the national context for each renewable. We thus constructed a variable for each renewable representing the fraction of installed capacity of each RES in each district relative to total capacity installed in Portugal (variable *national contribution of region for the RES*).

Through regression analysis, we estimate the net marginal impact that each of these personal and regional factors have on the perception of RES.

### 3.2. Empirical Study: Application to the Portuguese Case

#### 3.2.1. The Portuguese Case

In Portugal, between 2000 and 2018, the installed capacity for the production of electricity from RES increased from 4.8 GW to 14.1 GW, with an average annual growth of around 6%. In 2018, the hydroelectric component was responsible for 44.2% of the electricity produced, followed by wind production (41.2%), biomass (10.5%), photovoltaic (3.3%) and geothermal (0.7%). Observing the data by region, about 86% of the production took place in the North and Centre of the country, where the bulk of aerogenerators and most of the dams are located [53,54].

**Figure 1:**
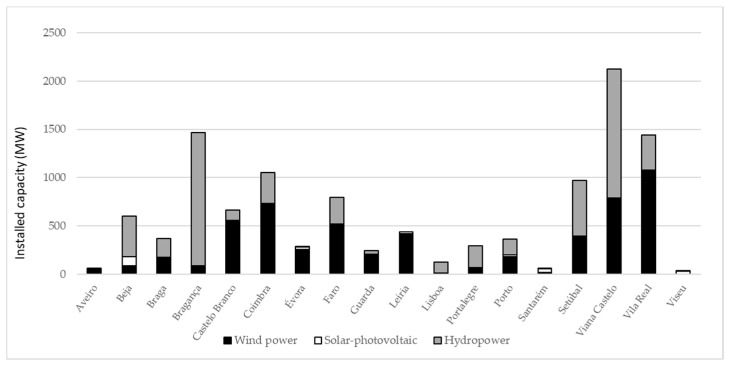
Renewables installed capacity by districts in mainland Portugal; Source: Authors’ own calculations based on data from 2013 accessed in http://e2p.inegi.up.pt/.

Regarding the geographical distribution of RES in mainland Portugal, there are regional differences in terms of installed capacity of RES. To better contextualize the case study at the time the data collection was carried out, Figure 1 presents the installed capacity of RES by district at the end of 2013 in mainland Portugal. In this paper, we focus our analysis on the three renewables that are more familiar to respondents: hydropower, wind power and solar-photovoltaic. A significant variability in installed capacity by district of those renewables is observable; renewables are almost non-existent in some districts, while others have a very significant presence of one, or more, RES. It should be noted that while the empirical analysis will use data from these districts in terms of renewable installed capacity, we aim to look beyond the specific districts to consider regional characteristics that can be applied in other contexts as well. This means that the data is specific to Portugal, but the approach can be applied elsewhere.

#### 3.2.2. Sample Characteristics

During the first half of 2014, questionnaires were collected from a national sample using a face-to-face approach and personal interviews, through a specialized polling company that contacted participants following a stratification based on gender, age and district of residence. Upon contact by the polling company, participation was voluntary. For the purpose of this paper, we have a sample of 1678 questionnaires (While there are 1678 complete questionnaires for the purpose of the complete regression analysis, we do not have information for some descriptive statistics within these 1678 questionnaires. Rather than reduce the number of observations for the descriptive and regression analysis to a common smaller sample, or report fewer sample characteristics, we opt to present descriptive statistics for the smaller subsamples for which there are responses but ensure a more complete sample for the regression analysis. The variables in Table 1 for which there is missing information concern the work situation, marital status, schooling and monthly electricity bill—this information is only relevant for sample description and was not used as the basis of the sample stratification nor is used, as mentioned, in the regression analysis. The fact that respondents were not forced to respond to all questions further enhances the voluntary participation nature of the questionnaire).

Table 1 reports socio-demographic sample characteristics. According to the values presented, we observe that: respondents are on average 48 years old; around 54% of respondents are employed or self-employed; about 40% hold at least an undergraduate degree; and respondents’ monthly electricity bill is, on average, 66.71 euros (note that the minimum wage at the time the questionnaires were collected amounted to 565.8 euros).

Table 2 reports respondents’ perceptions regarding environment and RES. We observe that respondents perceive the most significant environmental problems in Portugal as being climate change and the management of waste. It is noteworthy that almost all respondents are familiar with the three RES explored in this paper, but less with the other sources of renewable energy. Regarding proximity, 27% of respondents see a RES power plant daily (from home, work or during the daily commute). Most frequently, the source they see is wind power, either from their residence or during their daily commute.

## 4. Results

To model respondents’ opinions on RES environmental impacts as a function of location-related factors, a regression model is estimated and several covariates are considered, including two control variables for individual characteristics (*age* and *gender*). The explanatory variables characterize the relation of the respondent to the RES (*visibility of RES power plant, involvement*). The variable *visibility of RES* is a dichotomous variable that captures whether the respondent sees an RES power facility either from home, work or during the daily commute. The variable *involvement* is a dichotomous variable to indicate if the respondent is directly or indirectly benefited from RES, namely whether the respondent, a family member or an acquaintance worked at the time of the questionnaire or in the past in the renewable energy sector. The remaining explanatory variables characterize the region in terms of RES (*preponderance of the RES*, *national contribution of the region for the RES*) and are presented Section 3.1.

The regression model used is an Ordered Probit model. This is a multinomial model where the dependent variable is categorical and outcomes are ordered. There is an order in the responses in terms of the degree of support for a particular RES, but the outcome variable is not numerical but rather categorical. As presented by Cameron and Trivedi ([55], p. 519 and [56], p. 510), the Ordered Probit model is thus an appropriate model to analyse the data. Stata© 15 was used to estimate the model.

In Table 3, we present the estimation results of the Ordered Probit model for the perception of three renewables: hydropower, wind and solar photovoltaic (SPV). The presented results reveal interesting insights with respect to the effect of the presence of RES on respondents’ evaluation of the environmental impacts of each renewable. We can interpret a (statistically significant) positive coefficient of independent variables as indicative that, all other variables being equal, that particular variable increases the likelihood of a more positive perception of the friendliness of RES. On the contrary, a negative coefficient implies a negative relation between the variable and the perception of friendliness.

Analyzing the results for wind power (1), we observe that personal location-related factors, *visibility* and *involvement* variables, have a positive impact on the perception of friendliness of wind power. As for regional factors, having a higher fraction of wind power in the district decreases the probability of finding positive environmental impacts from wind power plants, all else being equal (*national contribution of region for wind*). When the primary renewable source in the district is hydropower, the perception of wind power is worse (*preponderance of hydropower* = 1); when SPV is the predominant renewable, the perception of wind power is better (*preponderance of SPV* = 1). These results suggest that perceptions of friendliness, in this case of wind power, depend on the relative importance of different renewables in the district of residence, with hydropower creating a negative spillover, and SPV a positive spillover on the perception of wind power.

Regarding hydropower (2), the visibility of power plants has a negative impact on the perception of that RES, whereas the involvement variable does not have a statistically significant coefficient. As for the importance of RES, the fraction of hydropower installed in the district has no incremental effect on respondents’ evaluation of the environmental effects of hydropower (*national contribution of region for hydropower*). Being a resident in a district where the main power source installed is SPV (*preponderance of SPV* = 1) or wind (*preponderance of wind* = 1), positively affects respondents’ judgements on the environmental effects of hydropower.

Results for SPV (3) show that respondents directly or indirectly involved with the RES tend to positively evaluate the environmental effects of SPV (*involvement)*. However, the regional contribution to the national production of SPV has not statistically significant impact. When respondents live in districts where either wind (*preponderance of wind* = 1) or hydric power (*preponderance of hydropower* = 1) are the predominant renewable source of energy, the perception of SPV is negatively impacted.

Finally, it should be stressed that the predicted probability of respondents perceiving hydropower, wind power, and solar photovoltaic as environmentally unfriendly (outcomes 1 and 2 in the 5-point Likert scale) is 9.1%, 0.7% and 0.7%, respectively. Thus, hydropower is considered more environmentally detrimental than the other energy sources.

## 5. Discussion

Increasing national demand for energy requires either the expansion of own energy production, or the increase in imports. Moreover, in the context of climate change and air pollution, the challenge to reduce CO_2_ emissions from the electricity production sector is a priority. As such, the increase in electricity production is increasingly demanding higher shares of renewable energy sources. The use of renewables contributes both to the use of endogenous sources of energy, and thus to energy security, and to the reduction in CO_2_ emissions, as long as they enter the energy mix in substitution of fossil fuels. However, the use of RES for electricity production is not environmentally impact free [18]. In addition, the negative impacts tend to be concentrated near the installation. As discussed by Sovacool [11], the question of how the impacts are distributed is crucial from an equity standpoint. Moreover, the social acceptance of additional power plants also depends on the perception of the equity of the decision.

To continue to promote RES for electricity generation in terms of expansion of existing projects and sources and development of new ones, policy-makers need to further the investment in renewables. For this, it is important to have citizen support in terms of public opinion and acceptability of new projects. Policy makers can devise communication strategies aimed at increasing the support of national citizens, but need to understand antecedents of perceptions towards renewables. Studies so far have taken two different views of public support. Generic perceptions of national residents have been analysed, while other studies have approached local residents and focused on very localized effects. Batel and Devine-Wright [16] argue there is a gap between documented national and local attitudes towards renewables partly due to whether the studies focus on more local or more generic impacts of renewables. In this context, some authors have attempted to bridge this gap by for example gauging generic acceptance through questionnaires for hypothetical locations close to respondents [17,57]. In our study, we took a different but complementary approach. We proposed to analyse of the antecedents of the social acceptance of RES using objective location-related factors, that is we elicit the perception of friendliness of the RES and partially explain it through respondent variables that are location-related. This approach does not replace existing studies, but rather complements them by introducing objectively measured variables that can partly explain why different individuals perceive specific RES differently, as a function of location-related factors. Using data from a questionnaire where the social acceptance is proxied by eliciting the perception of a particular RES’s environmental friendliness, we put this approach to the test. Overall, we find that location-related factors, either personal or regional, have statistically significant and non-negligible impacts on respondents’ perceptions. The empirical results show that respondents’ perceptions of the environmental impacts of three specific energy sources is significantly impacted by the respondent/a family member/an acquaintance’s work involvement with the RES under analysis. In addition, seeing a wind power plant daily has a marginal positive impact on the perception of wind power. By contrast, seeing a hydropower plant has a marginal negative impact. It is possible that local wind farms benefit from the documented effect in the literature where “energy technologies in the landscape can be ascribed as well a positive aesthetic value, as symbols of progress, modernity and development.” ([50], p. 91). As for the latter negative effect for hydropower, we can attribute this result to the specificities of hydropower in Portugal, which is the oldest RES present. It involves large-scale projects which had significant environmental impacts during the sitting phase (e.g., [26,58]), as such it is often the least supported of renewables in Portugal [49]. It is thus unsurprising that the population is less accepting towards hydropower.

As for regional characteristics of the RES under analysis, we also find statistically significant effects. Controlling for all other variables, the more wind power a district has in the national installed capacity, the less positively a respondent perceives wind power. We do not find an incremental effect of this variable for the other renewables.

In terms of regional location-related factors, we also observe different effects on perceptions depending on the dominant renewable in the district of residence. We find, for example, than when respondents live in districts where hydropower is the dominant renewable, the perception of the other renewables is less positive. As for SPV, the spillover effect is positive for the perception of wind and hydropower (i.e., in districts where SPV is the most important renewable, the other renewables are relatively less prominent, and are better perceived).

## 6. Conclusions

To promote the energy transition to less polluting and more sustainable energy production systems, renewables need to continue to be implemented and developed. However, electricity production through renewables is not impact-free and requires investment by public authorities, legitimized by public support. The opinion of national citizens depends on different factors, and on the energy mix of any country. Recently, empirical studies have attempted to move from abstract perceptions of renewables to situations that are more concrete, so as to bridge the so-called local-national gap in perceptions. The present study takes a complementary approach and attempts to explain individual perceptions as a function of location factors. Where the individual lives and works determines their experience, involvement and acquaintance with RES. We proposed to explain individual perceptions of friendliness through personal and regional variables, elicited either through a questionnaire or through RES databases. Understanding what antecedents condition perceptions in this manner can help policy makers create more effective communication strategies.

For the specific case study in Portugal, we find that the generic perception of friendliness of renewables is indeed conditioned by the individual’s location-related factors. While the statistical relation we observe is related to the specific case study, the approach we apply can be used elsewhere to better understand public perceptions, by uncovering more objective antecedents of the social acceptance of RES. This approach can thus be another tool for policy makers when designing energy policy and communication strategies.

## Figures and Tables

**Table 1:** Descriptive statistics: socio-demographic characteristics.

		Mean or Frequency (%)	Number of Responses
Gender	male	47.3%	1678
	female	52.7%	
Age		48.38	1678
Work situation	Unemployed	12.5%	1597
	Housework	2.6%	
	Student	6.3%	
	Retired	24.7%	
	Self-Employed	9.7%	
	Employed	44.2%	
Marital status	Married	63.4%	1644
	Divorced	5.8%	
	Single	26.0%	
	Widow	4.8%	
Schooling	Primary (years 1–4)	12.7%	1530
	Preparatory (years 5–6)	5.1%	
	Secondary (years 7–9)	13.3%	
	Post-secondary (years 10–12)	29.8%	
	Undergraduate	32.5%	
	Master	5.2%	
	PhD	0.9%	
	Other	0.5%	
Monthly electricity bill		€66.71	1423

**Table 2:** Descriptive statistics: environment and renewable energy sources (RES) perceptions.

		Frequency (%)
Environmental problems (indicate the 3 main problems facing Portugal)	Air pollution	48.5%
	Water pollution	48.4%
	Over-exploitation of natural resources	9.2%
	Decreased biodiversity	16.5%
	Climate change	45.9%
	Waste	48.2%
	Other	3.6%
Which of the following RES are you aware of?	Wind	99.0%
	Solar	96.2%
	Forest biomass	55.9%
	Geothermic	55.9%
	Hydropower	96.3%
	Wave	71.9%
Do you see a RES power plant from home, work or during commute?		26.9%


Note: Number of observations is 1678 for all variables.

**Table 3:** Ordered Probit estimation results by RES.

	(1)	(2)	(3)
VARIABLES	Wind	Hydro	Solar Photovoltaic
			
Visibility of RES power plant (1: yes 0: no)	0.137 *	−0.112 *	0.049
	(0.078)	(0.063)	(0.077)
Involvement (1: yes 0: no)	0.243 **	0.076	0.272 **
	(0.107)	(0.086)	(0.108)
Preponderance of wind (1: yes 0: no)		0.116 *	−0.402 ***
		(0.065)	(0.145)
Preponderance of hydropower (1: yes 0: no)	−0.236 ***		−0.433 ***
	(0.078)		(0.161)
Preponderance of SPV (1: yes 0: no)	0.291 **	0.394 ***	
	(0.142)	(0.122)	
National contribution of region for wind (%)	−2.496 ***		
	(0.794)		
National contribution of region for SPV (%)		−0.635	
		(0.563)	
National contribution of region for hydro (%)			−0.042
			(0.691)
Age	−0.003 *	−0.000	−0.004 *
	(0.002)	(0.002)	(0.002)
Gender (1: male)	0.036	0.047	0.017
	(0.066)	(0.055)	(0.067)
Prob (outcome 1)	0.004 ***	0.023 ***	0.005 ***
	(0.002)	(0.004)	(0.002)
Prob (outcome 2)	0.003 **	0.068 ***	0.002 **
	(0.001)	(0.006)	(0.001)
Prob (outcome 3)	0.016 ***	0.046 ***	0.022 ***
	(0.003)	(0.005)	(0.004)
Prob (outcome 4)	0.211 ***	0.416 ***	0.183 ***
	(0.010)	(0.012)	(0.010)
Prob (outcome 5)	0.766 ***	0.447 ***	0.788 ***
	(0.010)	(0.012)	(0.010)
Observations	1678	1678	1678
Wald-chi2	41.69	23.79	23.37

Notes: Dependent variable is the *perception of friendliness of RES* on a 1–5 scale 1: very unfriendly to 5: very friendly; independent variables are dichotomous categorical assuming 1 when the characteristic is present and 0 otherwise (except *age* and *national contribution* which are continuous in years and in percentage respectively); SPV stands for solar photovoltaic; robust standard errors in parentheses: significance level of 1% ***, 5% ** and 10% *.

## Data Availability

Raw data from the questionnaire is available from the authors upon request.

## Supplementary Materials

The following are available online at https://www.mdpi.com/1660-4601/18/2/806/s1: Supplementary Material: Questions from the questionnaire (translated into English from the original Portuguese questionnaire).
