# Supplementary material for: Explaining the Social Acceptance of Renewables through Location-Related Factors: An Application to the Portuguese Case
Source: Int J Environ Res Public Health. 2021 Jan 19;18(2):806. doi: 10.3390/ijerph18020806 (PMC7832858; doi:10.3390/ijerph18020806)
Supplement: Supplementary file 1 [file ijerph-18-00806-s001.pdf]

Explaining the Social Acceptance of Renewables through Location-Related Factors: An Application to the Portuguese Case

Supplementary material: Questions from the questionnaire (translated into English from the original Portuguese questionnaire)

Note: The full questionnaire included other questions, in particular several choice sets for a Discrete Choice Experiment and different sections depending on the renewable energy sources under analysis, and these were excluded here for parsimony.

Dear respondent,

We need your collaboration in a research project conducted by researchers of the University of Minho. Its main objective is the valuation of environmental impacts associated with each of the various renewable energy sources. The following questionnaire is anonymous and confidential. Please respond with the greatest sincerity possible.

It is very important that you fill out the questionnaire until the end.

Section I: Introductory Section

In this section we would like to have an idea of your familiarity with renewable energy.

1. What are the most important environmental problems in Portugal currently? (Indicate only 3)

- ☐ Climate change
- ☐ Atmospheric pollution
- ☐ Water pollution (rivers and ocean)
- ☐ Over-exploitation of natural resources
- ☐ Biodiversity decline (variety of animal and plant species)
- ☐ Waste
- ☐ Other

5. Which of the following renewable energies sources are you aware of?

- ☐ Wind Power
- ☐ Photovoltaic Power (solar)
- ☐ Biomass (forest remains)
- ☐ Geothermal Energy (heat of the earth)
- ☐ Hydropower (dams)
- ☐ Wave Energy
- ☐ Other ___________

6. What is your opinion on how environmentally friendly are these energy sources? (Indicate with an X).

	Very friendly	Somewhat friendly	Indifferent	Somewhat not friendly	Not friendly	I don’t know

Nuclear						
Hydropower (dams)						
Coal						
Natural gas						
Wind power						
Photovoltaic (solar)						
Geothermal (heat of the earth)						
Biomass (forest waste)						
Fuel oil (gas oil)						
Wave energy						

8. Do you work/have you worked in any technology of production of renewable energy?

☐ Yes ☐ No

8.1. If yes, specify in which one(s):

☐ Wind farm
☐ Photovoltaic farm (solar)
☐ Dam
☐ Biomass power plant

9. Do you know someone that works/worked in any technology of production of renewable energy?

☐ Yes ☐ No

9.1 If yes, specify in which one(s):

☐ Wind farm
☐ Photovoltaic farm (solar)
☐ Dam
☐ Biomass power plant

11. What is your monthly amount (average/approximately) of your electricity bill?

Value_______________ do not know ______

Section IV: Sociodemographic questions

30. Gender: ☐ Female ☐ Male

31. Marital Status:

☐ Married/ Facto Union
☐ Divorced
☐ Single
☐ Widower

32. Age: ____________

33. Situation in terms of employment:

- ☐ Unemployed
- ☐ Domestic
- ☐ Student
- ☐ Retired
- ☐ Self employed
- ☐ Worker as an employed person

34. Academic qualifications:

- ☐ 1st, 2nd, 3rd or 4th year (former primary instruction)
- ☐ 5th or 6th year (former preparatory cycle)
- ☐ 7th, 8th or 9th year (former 3rd, 4th and 5th lyceum year)
- ☐ 10th, 11th or 12th (former 6th and 7th lyceum year/introductory year)
- ☐ Bachelor or Degree
- ☐ Master
- ☐ Doctoral Degree (PhD)
- ☐ Other

37. Do you see a RES power plant from home, work or during daily commute?

☐ Yes ☐ No

37.1 If yes: which is the renewable energy source?

- Wind power ☐
- Hydropower (dam) ☐
- Biomass (forest remains) ☐
- Photovoltaic (sun) ☐

37.2 If yes: from where?

☐ Residence ☐ Work ☐ Daily commute

38. What is your municipality of residence? ___________________

THANK YOU VERY MUCH FOR YOUR COOPERATION!
